# Estimating the prevalence of poor-quality anti-TB medicines: a neglected risk for global TB control and resistance

**DOI:** 10.1136/bmjgh-2023-012039

**Published:** 2023-07-11

**Authors:** Patricia Tabernero, Paul N Newton

**Affiliations:** 1 Public Health Unit, Faculty of Medicine, Universidad de Alcalá, Alcalá de Henares, Spain; 2 Lao-Oxford-Mahosot Hospital-Wellcome Trust Research Unit, Vientiane, Lao People's Democratic Republic; 3 Medicine Quality Research Group, Centre for Tropical Medicine and Global Health, Nuffield Department of Medicine, University of Oxford, Oxford, UK; 4 Infectious Diseases Data Observatory (IDDO)/WorldWide Antimalarial Resistance Network (WWARN), Centre for Tropical Medicine and Global Health, Nuffield Department of Medicine, University of Oxford, Oxford, UK

**Keywords:** tuberculosis, epidemiology, pharmacology, public health, systematic review

## Abstract

**Objectives:**

Tuberculosis (TB) remains a major global public health problem, especially with the recent emergence of multidrug-resistant TB and extensively drug-resistant TB. There has been little consideration of the extent of substandard and falsified (SF) TB medicines as drivers of resistance. We assessed the evidence on the prevalence of SF anti-TB medicines and discussed their public health impact.

**Materials/methods:**

We searched Web of Science, Medline, Pubmed, Google Scholar, WHO, US Pharmacopeia and Medicines Regulatory Agencies websites for publications on anti-TB medicines quality up to 31 October 2021. Publications reporting on the prevalence of SF anti-TB drugs were evaluated for quantitative analysis.

**Results:**

Of the 530 screened publications, 162 (30.6%) were relevant to anti-TB medicines quality; of those, 65 (40.1%) described one or more TB quality surveys in a specific location or region with enough information to yield an estimate of the local prevalence of poor-quality TB medicines. 7682 samples were collected in 22 countries and of those, 1170 (15.2%) failed at least one quality test. 14.1% (879/6255) of samples failed in quality surveys, 12.5% (136/1086) in bioequivalence studies and 36.9% (87/236) in accelerated biostability studies. The most frequently assessed were rifampicin monotherapy (45 studies, 19.5%) and isoniazid monotherapy (33, 14.3%), rifampicin-isoniazid-pyrazinamide-ethambutol fixed dose combinations (28, 12.1%) and rifampicin-isoniazid (20, 8.6%). The median (IQR) number of samples collected per study was 12 (1–478).

**Conclusions:**

SF, especially substandard, anti-TB medicines are present worldwide. However, TB medicine quality data are few and are therefore not generalisable that 15.2% of global anti-TB medicine supply is SF. The evidence available suggests that the surveillance of the quality of TB medicines needs to be an integral part of treatment programmes. More research is needed on the development and evaluation of rapid, affordable and accurate portable devices to empower pharmacy inspectors to screen for anti-TB medicines.

**WHAT IS ALREADY KNOWN ON THIS TOPIC**

- Substandard and falsified (SF) medicines pose a severe global public health threat, as patients risk impaired therapeutic efficacy and/or adverse drug reactions.
- There has been little consideration of the extent of poor-quality tuberculosis (TB) medicines and their contribution to the high burden of multidrug-resistant TB and extensively drug-resistant TB.

**WHAT THIS STUDY ADDS**

- Sixty-five publications were identified, including a total of 7682 samples collected in 22 countries. Of those, 1170 (15.2%) failed at least one quality test. Medicines collected included 17 different active pharmaceutical ingredients (APIs) and 7 different coformulations.
- The curated data suggest an important issue with substandard anti-TB medicines but only a handful of quality surveys have focused on specific drugs and in particular countries.
- More information collected with standardised methodology is needed to compare between countries and regions. These data are not generalisable to suggest that 15.2% of the global anti-TB medicines supply is SF.

**HOW THIS STUDY MIGHT AFFECT RESEARCH, PRACTICE OR POLICY**

- Surveillance of the quality of TB medicines needs to be an integral part of treatment programmes and its link to TB drug resistance needs to be further studied to inform policy.
- Important insights into the relationships between poor quality medicines of reduced %API/dissolution, mg/kg body weight dosage variability and patient outcome are needed.
- More research on the development and evaluation of rapid, affordable and accurate portable devices will empower pharmacy inspectors for screening anti-TB medicines for those with %API and dissolution outside of reference limits.

## Introduction

Despite substantial improvements in the fight against tuberculosis (TB), it remains a major global public health problem. Approximately 10.6 million (95% CI 1.3 to 1.5) people are estimated to fall ill with TB each year and ~1.4 million (95% CI 1.3 to 1.5) HIV-negative people died of TB in 2021.^1^ Furthermore, HIV and then the COVID-19 pandemics have reversed years of progress made in the fight against TB.^2 3^

Short-course regimens of first-line drugs can cure 85% of patients and hence most deaths are preventable.^4^ Standard treatment of pulmonary drug-susceptible TB comprises a 6-month fixed-dose combination (FDC) regimen of four first-line drugs: isoniazid, rifampicin, ethambutol and pyrazinamide.^5 6^

Efficacious TB drug treatments were first developed in the 1940s but drug-resistant strains appeared rapidly after the introduction of streptomycin in 1943.^7^

FDCs are encouraged by the WHO and the International Union Against Tuberculosis and Lung Disease as a promising approach to simplify TB treatment, avoiding monotherapy and improving drug supply management.^8^ FDCs were expected to reduce the emergence of drug resistance.^8 9^

Multidrug-resistant TB (MDR-TB) and extensively drug-resistant TB (XDR-TB) are associated with lower cure rates and higher morbidity and mortality. MDR-TB is defined as resistance to isoniazid and rifampicin^8^ and XDR-TB as resistance to rifampicin, isoniazid and to at least one fluoroquinolone and one second-line injectable agent such as amikacin or capreomycin.^10^ Rifampicin-resistant TB (RR-TB) also requires treatment with second-line drugs.^11^ Medicines to treat resistant disease are expensive and may need to be given for at least 9 months and up to 20 months, coupled with counselling for adverse events.^11^ Globally, an estimated 3.8% (3.2–4.4) of new TB cases and 7.7% (5.8–9.7) of previously treated patients have MDR/RR-TB.^1 11 12^

Effectiveness of treatments is compromised by numerous factors, including poor patient adherence, that is especially problematic due to the long treatment duration, side effects, drug-drug interactions and lack of awareness of the dangers of monotherapy. Poor prescribing practices in many countries^13^ and interrupted drug supply due to financial constraints or political instability have led to inadequate treatment regimens with inappropriate choice of drugs, dose and duration.^14^ Furthermore, the private sector, often unregulated and fragmented, dispenses similar quantities of TB drugs as the public sector.^15^ These factors contribute to reducing patient’s quality of life, avoidable morbidity, treatment failure or even death, and engender emergence of drug-resistant strains.

An additional factor, of uncertain relative importance for both patient outcome and drug resistance, that has received scant attention is the quality of TB medicines that patients take. Poor-quality medicines include both substandard and falsified (SF) medical products. Substandard medicines (also called ‘out of specification’) are authorised medical products that fail to meet either their quality standards or specifications, whereas falsified medical products deliberately/fraudulently misrepresent their identity, composition or source.^16^

The presence of SF anti-TB medicines has been documented in a few surveys,^17–21^ but the majority of reports have focused on the reduced bioavailability of rifampicin in FDC,^22–24^ as it is especially dependent on the quality of the raw active pharmaceutical ingredient (API) and manufacturing procedures.^8^ In the early 2000s inadequate blood levels of the TB medicines were reported because of poor medicine quality rather than poor absorption.^8^ Many reviews on TB and resistance have been published,^25–31^ but the quality of medicines has only been referenced briefly.^10 13 32–36^

Whereas poor-quality medicines have been widely described for other diseases, there has been little consideration of the extent of poor-quality TB medicines and their contribution to the high burden of MDR-TB and XDR-TB.^37^ The global extent of poor-quality TB medicines is still unknown, and their epidemiology poorly understood.

We therefore reviewed the available data on the quality of anti-TB medicines, analysed the epidemiology to better understand the limitations of the existing data and discuss their implications for TB control, morbidity and mortality and potential development and spread of TB resistance.

The data are curated and visualised within the Medicine Quality Scientific Literature Surveyor^38^ that includes a comprehensive, open-access, global database on the quality of anti-TB medicines reports.

## Methods

### Search strategy

A systematic review was conducted of scientific and lay reports on anti-TB medicine quality, using Web of Science, Medline, Pubmed, Scopus, Google, Google Scholar, WHO, US Pharmacopeia (USP), and Medicines Regulatory Agencies (MRA) websites.

Search included any published report, without an inclusion start date, up to 31 October 2021 in English, French and Spanish. Search terms included the terms ‘falsified’, ‘substandard’, ‘counterfeit’ and other commonly used terms such as ‘fake’ or ‘spurious’ or ‘quality’, together with ‘anti-TB’ or ‘TB medicines’ (online supplemental table 1). Manual searches of the reference lists in the included articles were also performed.

10.1136/bmjgh-2023-012039.supp1 Supplementary data

### Eligibility criteria

The inclusion criteria were of any study describing in vivo or in vitro tests to determine anti-TB medicine quality, assays to determine quality, articles about seizures, recalls and confiscations of anti-TB medicines; case reports or articles describing adverse events or patients not responding to anti-TB treatments where quality was questioned.

We included studies that surveyed the quality of TB drugs in one or more locations (hereafter ‘quality surveys’), that compared the pharmaceutical equivalence of different brands of a given TB drug (hereafter ‘bioequivalence studies’), studies describing techniques to determine the quality of TB drugs (hereafter ‘laboratory assembled collections’), studies aiming to predict the stability profile of a medicine (hereafter ‘accelerated stability assays’) and recalls, seizures or case reports by national medicines regulatory authorities (NMRA) (hereafter ‘case reports/NMRA seizures’).

Studies with results from several countries or locations are included under each specific country/location. A ‘data point’ is defined as a specific location where medicines were collected for quality analysis, at a given time during a given study.

No time restrictions were applied for the systematic search of the literature.

General discussions over sampling methodology and pharmaceutical legislation (eg, on the regulatory framework surrounding SF TB treatment) and reviews of the literature on various aspects of TB medicines were also included in the database for reference but are not included in the analysis of this review.

The exclusion criteria were studies with results for a whole region or a whole class of TB drugs, without specific country or location data. We also did not include reports for ciprofloxacin and co-amoxiclav as, although there are many reports of SF products, they are included in the parallel review of the quality of antibiotics.^39^ We did not include reports of quackery and use of SF TB medicines without an objective evidence base of efficacy. Neither the quality of BCG vaccines or diagnostic tests for TB were included.

### Key variables and definitions

Anti-TB medicine quality failure rates were extracted into a database from each report, including data on packaging, content of API, disintegration, dissolution, microbiology and accelerated stability results when available. Poor quality medicines classified as falsified, substandard or degraded by the reports’ author’s definitions were tabulated and interpreted in relation to current WHO definitions.^40^ Samples that failed chemical assays, but without detection of wrong active ingredients and without packaging analysis, are classified as substandard or falsified (SorF) and not as either falsified or substandard as this distinction cannot be reliably made without reference to the packaging or regulatory status of the sample.^41^ However, samples that contained the wrong API or no API but without packaging analysis were assumed to be falsified. There is a small risk of misclassification of such samples, as falsified when they are actually substandard, due to gross manufacturing errors.^42 43^ Samples that did not fail chemical and packaging tests (when these were done) are considered as good quality.

### Data collection

Information within each publication was manually extracted and entered in a database constructed using MS Access 2010, developed in collaboration with the informatics team at IDDO (https://www.iddo.org/mq-scientific-literature-surveyor). Information extracted included: publication type (eg, report, original research article), year of publication, publisher, sampling type, location (country and city, where available) and type of outlet where samples were collected, total number of samples collected, API/API combination name, number of samples failing medicine quality test(s) and the techniques used to analyse samples.

### Analysis and reporting

Data were extracted and statistical analyses were conducted in Microsoft Excel 365 and Stata (V.11.2, Stata Corp, College Station, Texas, USA). This review follows the Preferred Reporting Items for Systematic Reviews and Meta-Analyses (PRISMA) guidelines.

### Risk of bias assessment

Since there are no standardised methods to assess equivalence, analysis technique, lay press and case reports publications, their risk of bias was not addressed. Publication bias should be taken into consideration.

### Patient and public involvement

Patients or the public were not involved in the design, or conduct, or reporting, or dissemination plans of our research.

### Ethics approval

No ethical approval was sought for this study and no patient identifiable data were used.

## Results

### Types and timing of publications

**Figure 1:**
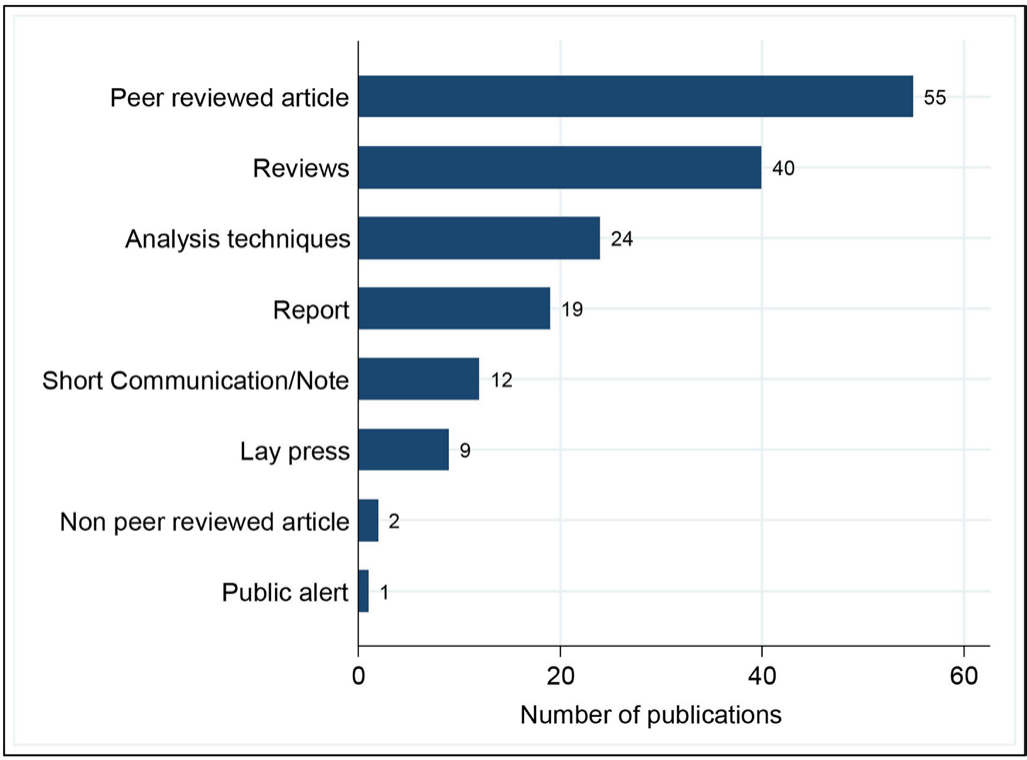
Types of publications related to the quality of medicines for tuberculosis.

A total of 530 publications were screened by title and abstract, 507 gathered through electronic searches and 23 additional publications identified through manual reference screening and other sources such as lay press, NGOs, WHO and US Pharmacopeia Medicines Quality Database reports. See figure 1, PRISMA statement and flow chart of papers in the online supplemental material.

After removal of duplicate reports and articles excluded for eligibility, 278 full-text publications were reviewed and 162 were included. Other articles were excluded as they were describing surveys, techniques to determine the quality of drugs and seizures of other classes of medicines (n=116).

A third of the articles were primary research peer-reviewed papers (55, 33.9%), followed by peer-reviewed reviews or compilations of articles (40, 24.7%) and those describing laboratory assembled collections for TB medicines (24, 14.8%). Reports from international organisations accounted for 19 (11.7%) of the publications. Other types of articles included short communications (12, 7.4%), lay press articles (9, 5.5%), two non-peer-reviewed articles (1.23%) and one public alert (0.6%) (figure 1). No PhD theses about TB medicine quality problems were found.

The first publication we found reporting poor-quality TB medicines was dated from 1906,^44^ describing fake anti-TB nostrums, with no other publications found until 1968.^45 46^

**Figure 2:**
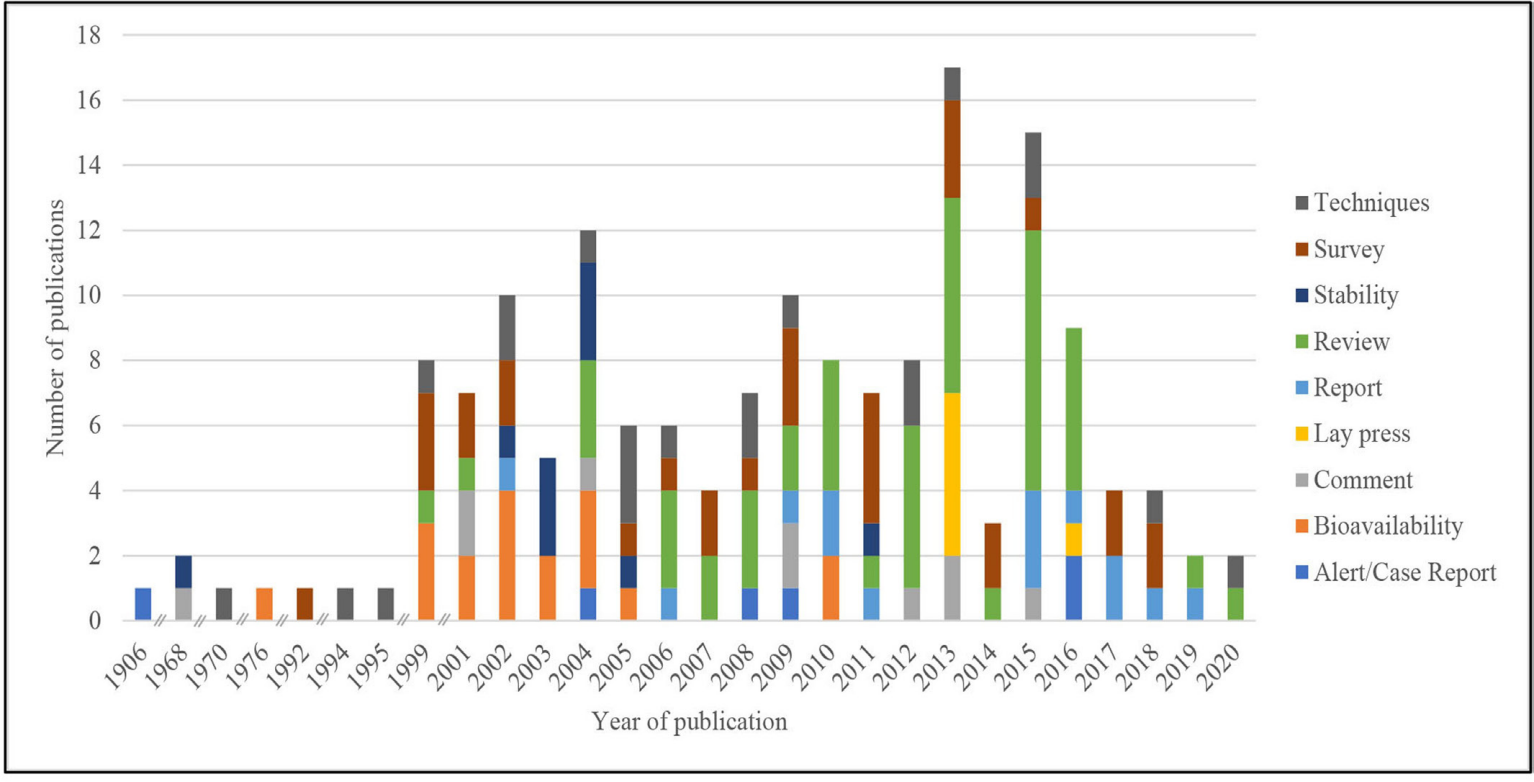
Number of publications over time related to the quality of medicines for tuberculosis.

Most of the publications were published since 1999 (154, 95.1%) with 17 (10.5%) reports being published in the year 2013. The most recent papers were from 2020 (2, 1.2%) (figure 2). Although reports were published in 70 different journals, 26 (16.5%) were published in the *International Journal of TB and Lung Disease* (https://www.theunion.org/what-we-do/journals/ijtld).

Of the 162 publications included in the database, 65 (40.1%) described one or more TB quality surveys in a specific location or region with enough information to yield an estimate of the local prevalence of poor-quality TB medicines; 231 records/data points of medicine samples identified in these 65 publications were included in the analysis.

Of the 65 publications, 7 publications were removed from the analysis as they included an unspecific number of samples; 6 had unknown numbers of samples collected^47–52^ and 1 publication reported the diversion of 2 million doses that were not analysed,^53^ leaving a total of 58 publications included in the quantitative analysis. Publications were then categorised by sampling methodology and study type. Sampling categories were not mutually exclusive. As a few studies were included in multiple sampling categories, the number of publications adds to more than 100% (n=67).

Half of the reports described quality surveys (33, 49.2%), followed by bioequivalence studies (17, 25.3%). Ten (14.9%) studies described accelerated biostability assays. Other studies included in the quantitative analysis were five laboratory assembled collection reports and two case reports/NMRA seizures (7.5% and 2.9%, respectively).

All publications have been mapped on the Infectious Diseases Data Observatory Medicine Quality Surveyor system and data are downloadable here^38^ (https://www.iddo.org/mqsurveyor/%23anti-TB).

### Type of medicines and geographical data

Medicines collected included 17 different APIs and 7 different coformulations. The most frequently assessed were rifampicin monotherapy in 45 studies (19.5%) and isoniazid monotherapy in 33 (14.3%), rifampicin-isoniazid-pyrazinamide-ethambutol FDC in 28 studies (12.1%) and rifampicin-isoniazid in 20 (8.6%). The median (IQR) number of samples collected per study was 12 (1–478). The content of the APIs was measured in 52 (80.0%) studies.

In total, 7682 samples were collected in 22 countries. Of those, 6255 were from quality surveys in 17 countries and 1086 samples were from equivalence studies in 6 countries.

The majority of data points were in India with 92 (41.4%), 20 (9.0%) in Kenya and 16 (7.2%) in Kazakhstan (table 1). The country where samples were collected was not specified for 985 (12.8%) samples and 11 (4.9%) data points (table 1).

**Table 1:** Failure rate per country in quality surveys, bioequivalence studies, accelerated biostability studies, laboratory assembled collections and case reports/MRA seizures

Countries	Accelerated biostability studies			Bioequivalence studies			Quality surveys			Laboratory assembled collections			MRA seizure			Total			
	N	n	Failure rate %	N	n	Failure rate %	N	n	Failure rate %	N	n	Failure rate %	N	n	Failure rate %	No of data points	N	n failed	Failure rate %
**Africa**				**627**	**65**	**10.4**	**122**	**33**	**27**							**45**	**749**	**98**	**13.1**
Botswana							13	4	30.8							3	13	4	30.8
Kenya							42	5	11.9							20	42	5	11.9
Nigeria				9	5	55.6	50	22	44.0							6	59	27	45.8
South Africa				618	60	9.7										13	618	60	9.7
Zambia							17	2	11.8							3	17	2	11.8
**Americas**	**18**	**0**	**0.0**	**18**	**18**	**100.0**	**18**	**0**	**0.0**							**5**	**54**	**18**	**33.3**
Mexico				18	18	100.0	18	0	0.0							2	36	18	50.0
USA	18	0	0.0													3	18	0	0.0
**Asia**	**218**	**87**	**39.9**	**436**	**53**	**12.2**	**5082**	**732**	**14**	**20**	**1**	**5.0**	**2**	**2**	**100.0**	**145**	**5758**	**875**	**15.2**
Armenia							42	4	9.5							5	42	4	9.5
Azerbaijan							31	3	9.7							5	31	3	9.7
Cambodia							117	31	26.5							2	117	31	26.5
India	206	87	42.2	362	6	1.7	3484	377	10.8	12	0	0.0	2	2	100.0	92	4066	472	11.6
Indonesia				74	47	63.5	71	4	5.6							4	145	51	35.2
Kazakhstan							842	176	20.9							16	842	176	20.9
Myanmar/Burma							13	1	7.7							1	13	1	7.7
Pakistan										8	1	12.5				2	8	1	12.5
Thailand	12	0	0.0				198	50	25.3							5	210	50	23.8
Uzbekistan							45	6	13.3							5	45	6	13.3
Vietnam							239	80	33.5							8	239	80	33.5
**Europe**				**5**	**0**	**0.0**	**113**	**6**	**5.3**	**18**	**0**	**0**				**16**	**136**	**6**	**4.41**
Republic of Belarus							60	4	6.7							5	60	4	6.7
Switzerland										4	0	0.0				1	4	0	0.0
Ukraine							53	2	3.8							4	53	2	3.8
UK				5	0	0.0				14	0	0.0				6	19	0	0.0
Unstated							920	108	11.7				65	65	100.0	11	985	173	17.6
**Total**	**236**	**87**	**36.9**	**1086**	**136**	**12.5**	**6255**	**879**	**14.1**	**38**	**1**	**2.6**	**67**	**67**	**100.0**	**222**	**7682**	**1170**	**15.2**

MRA, Medicines Regulatory Agency.

Of the 7682 samples collected, 1170 (15.2%) failed at least one quality test, 6.7% (78) were classified as falsified, 22.6% (265) as substandard and 70.7% (827) as SorF since packaging analysis was not performed (table 2 and online supplemental table 2).

**Table 2:** Quality of medicines per API in included quality surveys, bioequivalence studies, accelerated biostability studies, laboratory assembled collections and case reports/MRA seizures

Medicine name	Accelerated biostability studies			Bioequivalence studies			Quality surveys			Laboratory assembled collections			Case reports/MRA seizure			Total		
	N	n	Failure rate %	N	n	Failure rate %	N	n	Failure rate %	N	n	Failure rate %	N	n	Failure rate %	N	n failed	Failure rate %
Amikacin							224	57	25.4							224	57	25.4
Cycloserine	149	57	38.3				150	54	36.0							299	111	37.1
Doripenem	6	0	0.0				0	0								6	0	0.0
Ethambutol	4	2	50.0	113	0	0.0	623	100	16.1				1	1	100	741	103	13.9
Ethionamide				1	0	0.0	86	12	14.0							87	12	13.8
Gatifloxacin/Ciprofloxacin							0	0					65	65	100	65	65	100.0
Imipenem-Cilastatin	6	0	0.0				0	0								6	0	0.0
Isoniazid	1	0	0.0	197	0	0.0	1472	142	9.6							1670	142	8.5
Isoniazid-Ethambutol	9	7	77.8				5	2	40.0							14	9	64.3
Kanamycin							80	4	5.0							80	4	5.0
Levofloxacin							161	27	16.8	4	1	25.0				165	28	17.0
Meropenem	18	0	0.0				5	0	0.0							23	0	0.0
Moxifloxacin							40	0	0.0							40	0	0.0
Norfloxacin							6	0	0.0							6	0	0.0
Ofloxacin				9	5	55.6	145	31	21.4	4	0	0.0				158	36	22.8
Prothionamide							100	0	0.0							100	0	0.0
Pyrazinamide	1	0	0.0	195	0	0.0	733	65	8.9							929	65	7.0
Rifampicin	1	0	0.0	243	100	41.2	2025	337	16.6							2269	437	19.3
Rifampicin- Isoniazid- Ethambutol	9	7	77.8	44	0	0.0	69	26	37.7							122	33	27.0
Rifampicin-Isoniazid	6	3	50.0	26	8	30.8	169	3	1.8	7	0	0.0	1	1	100	209	15	7.2
Rifampicin-Isoniazid-Pyrazinamide	3	2	66.7	83	21	25.3	39	1	2.6	14	0	0.0				139	24	17.3
Rifampicin-Isoniazid-Pyrazinamide- Ethambutol	23	9	39.1	111	2	1.8	100	6	6.0	9	0	0.0				243	17	7.0
Rifampicin-Isoniazid-Pyrazinamide- Ethambutol- Pyridoxine							1	0	0.0							1	0	0.0
Streptomycin				64	0	0.0	22	12	54.5							86	12	14.0
Total	236	87	36.9	1086	136	12.5	6255	879	14.1	38	1	2.6	67	67	100	7682	1170	15.2

MRA, Medicines Regulatory Agency.

In quality surveys 879 (14.1%) samples failed at least one quality test out of 6255 collected, 136 (12.5%) samples out of 1086 in bioequivalence studies and 87 (36.9%) samples of 236 in accelerated biostability studies.

The highest proportion of substandard anti-TB medicines was observed in the Americas, although it only included one study of the bioavailability of co-formulated rifampicin-isoniazid-pyrazinamide medicines in Mexico and one accelerated biostability study of doripenem, imipenem-cilastatin and meropenem in the USA. Asia had the largest number of samples collected, 5758 samples in 11 countries with an overall failure rate of 15.2%. Failure rate ranged from 7.7% in Myanmar to 33.5% in Vietnam and 35.2% in Indonesia. Total samples collected in Africa accounted for 749 in five countries, with a 13.1% failure rate; Nigeria had the highest failure rate of 45.8%.

Thirteen publications specified if the failing samples had lower or higher amounts of the active substance in relation to predefined cut-offs. For rifampicin, 132 samples (30.2%) had lower amounts of API, followed by cycloserine (54, 48.6%), pyrazinamide (25, 38.5%), isoniazid (16, 11.3%), levofloxacin (9, 32.1%), ethambutol (7, 6.8%) and kanamycin (4, 100%). Higher amounts of API were found in 20 (4.6%) rifampicin samples, 11 (10.7%) ethambutol, 7 (4.9%) isoniazid and 5 (7.7%) pyrazinamide samples.

High performance liquid chromatography (HPLC) was conducted in 27 (51.9 %) studies, either alone or coupled with UV-spectrophotometry, calorimetry (DSC) or Fourier transform infrared spectroscopy. Three bioavailability studies conducted HPLC and measured drug levels (or its metabolites) in urine rather than serum.

Minilab thin layer chromatography (TLC) was performed in 7 (10.8%), either alone or coupled with an additional technique such as Raman, HPLC or LC with UV spectrophotometry.

Four studies (6.1%) used ultraviolet spectrophotometry alone to determine the content of the API.

Other methods used to analyse the quality of the medicines included: fluorimetry, direct analysis in real time coupled to a compact single quadrupole mass spectrometer (DART-QDa-MS-DART-QTOF), an ultra-fast UPLC-UV method and liquid chromatography, coupled with mass spectrometry (LC-MS). Ten studies (15.4%) did not specify the tests conducted to determine the API content.

Dissolution testing was included in 25 studies (38.5%), and of those studies only 5 included disintegration testing.

Seventeen (26.2%) studies included physical inspection and packaging inspection was specifically mentioned in only seven studies (10.8%).

A third of the publications (25, 38.5%) specified the cut-off followed to assess if the medicine met quality standards. Five publications mentioned using an 85%–115% API cut-off, four used a <80% limit, four publications 90%–110% and another two a 95%–105% cut-off. Ten (15.4%) reports specified using pharmacopoeial methods, three of them mentioned using the British Pharmacopoeia, another three the USP and one using the Indian Pharmacopoeia. The other 30 (46.1%) reports did not specify which pharmacopoeial method was followed.

### Quality surveys

Of the 33 publications describing surveys in 17 countries, 28 (84.8%) were convenience surveys and 5 (15.2%) used random sample location selection. Surveys accounted for 141 (61.0%) individual medicine samples of the total 231 data points.

India yielded the largest number of reports with 14 (34.1%), followed by Kenya (5, 12.2%) and Indonesia, Kazakhstan, Nigeria and Vietnam with 2 (4.8%) reports available for each country. Other countries that had one (2.4%) publication describing anti-TB medicine quality surveys included: Armenia, Azerbaijan, Botswana, Cambodia, Mexico, Myanmar, Republic of Belarus, Thailand, Ukraine, Uzbekistan and Zambia. Three surveys (9.5%) did not state the country of sample collection (table 1).

A third of surveys (10, 29.4%) stated that they sampled only private outlets, 20.6% (7) specified that samples were collected in public hospitals and 26.5% (9) reports did not specify which results referred to private or public outlets. Three (8.8%) surveys obtained the samples from the distribution agent or wholesale pharmacy, two (5.9%) from the manufacturer and one (2.9%) report sourced the medicines from WHO and another one from a private clinic. The type of outlets from which medicines were sampled was not specified in one survey report (2.9%).

The highest reported failure rate was for streptomycin (54.5%), followed by co-formulated isoniazid-ethambutol (40.0%) and co-formulated rifampicin-isoniazid-ethambutol (37.7%). Medicines with higher failure rates also included cycloserine (36.0%), amikacin (25.4%) and ofloxacin (21.4%). Levofloxacin, rifampicin and ethambutol monotherapy had similar failure rates (16.8, 16.6 and 16.1%, respectively). Only meropenem, moxifloxacin, norfloxacin, prothionamide and co-formulated rifampicin-isoniazid-pyrazinamide-ethambutol-pyridoxine had zero failure rates (table 2).

We found no data points for bedaquiline, clofazimine, delamanid, linezolid, p-aminosalycilic acid, pretomanid and terizidone.

### Bioequivalence studies

Seventeen (25.4%) publications described bioequivalence studies including 39 (16.9%) individual medicine data points in the database.

Twelve studies described bioavailability of anti-TB medicines in vivo, three in vitro alone and two studies included both in vivo and in vitro methodology. Four of the bioavailability studies compared analysis of urinary excretion data versus plasma concentration time profiles.

Of the bioavailability studies seven described the API content, and one included accelerated stability assays.

Bioavailability data points included 19 from India (48.7%), followed by 13 (33.3%) from South Africa, 2 (5.1%) from Indonesia, and Mexico and Nigeria had 1 (2.6%) each. The UK had three data points (7.7%) (table 1).

Of the 1086 samples analysed, 136 (12.5%) failed the bioavailability tests conducted. Of the failing samples, 100 (73.5%) were rifampicin monotherapy tablets and 53 of those were samples classified as substandard with poor bioavailability collected in South Africa. Other poor-quality samples included 21 samples of rifampicin-isoniazid-pyrazinamide FDC, 8 samples of rifampicin-isoniazid FDC, 5 ofloxacin samples and 2 rifampicin-isoniazid-pyrazinamide-ethambutol FDC samples (table 2).

Bioavailability studies included samples obtained from the manufacturing company (18, 46.2%) or a distributor (13, 33.3%). Samples collected from pharmacies, either public hospitals or private pharmacies and clinics accounted for 10.3% (4). Four data points (10.3%) did not specify sample origin.

### Accelerated bioavailability studies

Ten publications described the stability of anti-TB medicines under accelerated conditions, with eight in India, one in Thailand and one in the USA.

All except two studies were conducted between 2003 and 2005. One study conducted in 1968^45^ studied the stability of cycloserine (149 samples) and one in 2011 studied the stability of doripenem, imipenem and meropenem.^54^

The overall failure rate was 36.9%, varying from 77.8% failure rate for both rifampicin-isoniazid-ethambutol FDC and isoniazid-ethambutol FDC, respectively, 66.7% for rifampicin-isoniazid-pyrazinamide FDC, and 50% for both ethambutol in single form and rifampicin-isoniazid FDC. Rifampicin-isoniazid-pyrazinamide-ethambutol FDC had a similar failure rate to cycloserine (39.1% and 38.3%, respectively). Other medicines that had zero failure rate included doripenem, meropenem, co-formulated imipenen-cilastatin, rifampicin, isoniazid, and pyrazinamide monotherapy.

### Research on methods assessing TB drug quality

Although 25 publications were found describing different techniques for analysis of anti-TB monotherapy and FDC tablets and capsules, only 9 were eligible to be included in the quantitative analysis, as others were validation methods and techniques with no specific medicine quality data included (online supplemental table 3).

Four publications described in vitro methodology for assessing the bioavailability and bioequivalence of TB drugs, and the effect of food on rifampicin. One publication used a calorimetric method to screen for the presence of the less active diastereomeric (R, S) forms of ethambutol dihydrochloride^55^ and one described a chemiluminescent method for pyrazinamide.^56^ Seven publications described chromatographic techniques including rapid screening of pharmaceuticals by TLC. One publication described the colorimetric analysis of 14 anti-TB FDCs.^57^

One publication described a disintegration method for isoniazid^58^ and another three described dissolution assays. Two articles described paper analytical devices for fast field screening of antimicrobial medicines and anti-TB pharmaceuticals.^59 60^ Five reports described spectroscopic techniques from low-cost optical spectrometers^61^ to near-infrared spectroscopy and multivariate calibration to measure the dissolution^62^ (online supplemental table 3).

### Case reports

A 1906 publication from *The New York Times* described fake TB ‘nostrums’.^44^ Three reports described the presence of TB medicines as cryptic wrong ingredients in falsified medicines.^63–65^ Two publications described the analysis of 192 blister packs labelled as antimalarial artemisinin-based combination therapy seized in sub-Saharan Africa.^63 64^ Sixty-five (33.8%) of those samples contained either ciprofloxacin alone or a combination of chloramphenicol, ciprofloxacin, sildenafil and gatifloxacin (table 3).

**Table 3:** Summary of seizures; MRA recalls and case reports of TB medical products with wrong active ingredients

Title	First author/publisher	Year	Country	Reference
Fake consumption cures. Doctors call patent and proprietary medicines harmful	*The New York Times*	1906	USA	44
Matrix of Drug Quality Reports in USAID-assisted Countries – ‘vials labelled as ceftazidime (Fortum) injection re-used from hospital waste and adulterated with streptomycin’	Primo-Carpenter J; The United States Pharmacopeial Convention Inc.	2004	Vietnam	65 & Power G.
Database on the incidents of Counterfeit Medicines in the WHO-SEA Region	South East Asian FIP-WHO Forum of Pharmaceutical Associations	2004	India	www.searpharmforum.org
Substandard medicines in resource-poor settings: a problem that can no longer be ignored	Caudron J M *Tropical Medicine & International Health*	2008	Chad	48
South Africa withdraws TB drugs because of quality fears	Sidley P *British Medical Journal*	2008	South Africa	49
A Small Number of Bottles of the Antibiotic Rofact (Rifampin) May Contain a Different Drug	Health Canada; Government of Canada	2009	Canada	https://recalls-rappels.canada.ca/en/alert-recall/archived-small-number-bottles-antibiotic-rofactr-rifampin-may-contain-different-drug
WHO suspends TB drugs from big India supplier on quality fears	Hirschler B Thomson Reuters	2016	India	https://uk.reuters.com/article/us-india-pharmaceuticals-svizera/who-suspends-tb-drugs-from-big-india-supplier-on-quality-fears-idUKKCN0WK2HE?feedType=RSS&feedName=healthNewsMolt
In Côte d’Ivoire. two million TB drugs financed by The Global Fund sold in street markets: OIG	Braquehais S Aidspan OIG	2016	Côte d’Ivoire	https://aidspan.org/
Fingerprinting of falsified artemisinin combination therapies via direct analysis in real time coupled to a compact single quadrupole mass spectrometer	Bernier M C Analytical Methods. The Royal Society of Chemistry	2016	Sub-Saharan Africa	63
Triboelectric Nanogenerator (TENG) Mass Spectrometry of Falsified Antimalarials	Bernier M C Rapid communications in mass spectrometry RCM	2018	Sub-Saharan Africa	64

MRA, Medicines Regulatory Agency; TB, tuberculosis.

One lay press report described the discovery of vials labelled as ceftazidime (Fortum) injection reused from hospital waste and adulterated with streptomycin.^65^

In a reported 2009 recall from the Government of Canada, bottles of the medicine Rofact (rifampin), were erroneously filled with clonazepam 0.5 mg.^52^

Other recalls/case reports/NMRA seizures included a lay press report describing the diversion of TB medicines financed by The Global Fund sold in local markets in Côte d’Ivoire.^53^ One publication reporting ‘over concentration’ of TB drugs in Chad (unpublished results from Réseau Médicaments et Developpement).^48^ Antib-4 (pyrazinamide-ethambutol-isoniazid- rifampicin) and Ebsar (isoniazid-rifampicin) manufactured in India by Rusan Pharma were recalled in South Africa because of the presence of lower active ingredients than specified.^49^ A report from the South-East Asian FIP-WHO Forum of Pharmaceutical Associations described falsified Reinex 450 and Clombutol (anti-TB medicines) in India in 2003.^66^ Manufacturing of TB medicines was suspended at Svizera Labs, a major supplier to developing countries, in Mumbai, India, because of unreliable manufacturing standards^67^ (table 3).

## Discussion

Effective diagnosis and treatment of TB saved an estimated 74 million lives between 2000 and 2021^1 11 68^ and are among the most cost-effective health interventions.^26^ Despite these advances TB remains a global threat, especially with the emergence of MDR-TB and XDR-TB during the past decade.^3 10^ However, there is little awareness of poor-quality anti-TB medicines and the threat they may pose to individual patients, the control of TB and the rise of MDR-TB and XDR-TB.

The data reviewed here suggest that there are issues with the quality of TB medicines, but the paucity of data do not allow global or national estimates. Data reporting on TB medicine quality are few and many are of poor quality. Zabala *et al* analysed the publicly accessible survey data on the quality of ciprofloxacin and co-amoxiclav finding median (IQR) failure frequencies of 3% (0%–8%) and 21.3% (5.8%–38.6%), respectively.^39^

Of the 202 countries with TB epidemiology data,^1 11^ published information on anti-TB medicine quality was publicly available from only 22 (10.9%). Of the 30 TB high-burden countries that collectively account for 86% of patients with TB, reports on TB drug quality were available from 11 (36.6%).

India had the largest number of reports (29, 43.3%) followed by Kenya (5, 7.5%) and South Africa (4, 6.0%). Indonesia, Nigeria, Thailand and Vietnam had two (3.0%) reports each available. Cambodia, Myanmar, Pakistan and Zambia had only one (1.5%) report available each. Information on anti-TB medicine quality in the other 19 high-burden countries is lacking, at least in the public domain.

Furthermore, no reports from Bangladesh, China, the Democratic Republic of the Congo and the Philippines were found, and they are four of the eight countries, together with India, Indonesia, Nigeria and Pakistan that accounted for more than two-thirds of the global total incident TB cases in 2021.^1 11^

Only two reports were available from the Americas and the available information from Europe is sparse.

The lack of standardisation of reporting is also a serious limitation. Results are frequently not broken down by country and/or medicine. The number of samples is commonly not representative, and the small sample size per survey limits the interpretation of the results. Randomisation, the gold standard in estimation of prevalence, was used in only 5 (15.2%) quality surveys. Additional data on TB medicine quality that are not in the public domain are likely to be held by medicines regulatory authorities, the pharmaceutical industry and vertical TB control programmes.

There is a small risk of misclassification of samples that contained wrong API or no API as falsified when packaging analysis was not assessed, as they may be substandard, due to gross manufacturing errors.^42 43^ Furthermore, there is little information available to distinguish substandard medicines due to errors in factory production from those due to degradation (eg, due to postproduction inappropriate storage).

Bioequivalence studies against reference formulations are conducted to assure quality and are mandated by WHO as part of the prequalification programme for registration of rifampicin containing FDCs. Almost 40% of the samples collected in the retrieved studies focused on the quality of rifampicin in single-dose combinations and FDCs (in combination with isoniazid, pyrazinamide and ethambutol). Reduced rifampicin dissolution, absorption and hence bioavailability have been well described. Rifampicin has pH-dependent solubility, and particle size, formulation and changes in the crystalline structure during manufacture can alter its bioavailability, risking substandard products.^9 10 19 69–71^

Poor rifampicin biovailability in FDC risks low exposure of TB bacteria to rifampicin, engendering resistance to both rifampicin and the partner drugs in the FDC.^22–24^ High doses of rifampicin may produce thrombocytopenia, influenza-like syndrome, haemolytic anaemia and acute renal failure whereas large doses of isoniazid may result in hepatotoxicity. This is particularly important when medicines in tablets are being split in halves and thirds. Two studies described that the APIs isoniazid, pyrazinamide, ethambutol and rifampicin may be not uniformly distributed in the split proportions thus risking overdosing or underdosing.^72 73^ This is particularly important for medicines for children for whom drug bioavailability tends to be more variable than in adults.^74^ No reports were found studying FDCs for children. Body weight band-based dose optimisation has been suggested to help achieve optimal drug exposures and limit pharmacokinetic variability.^74^

Surveillance of antituberculous medicine quality is impeded by insufficient laboratory capacity to assess bioavailability. Simple assays to quantify active ingredients of anti-TB drugs should be deployed to empower pharmacy inspectors. New paper test cards are a promising approach to determine the presence of APIs qualitatively but they are not designed to detect substandard medicines nor falsified medicine containing the correct stated API.^59^ The Global Pharma Health Fund (GPHF) has developed relatively inexpensive field test kits, the GPHF-Minilab, for rapid detection of falsified medicines, that cover a wide range of antimycobacterials.^75^

High TB burden countries are mainly categorised in the climate zone IV region.^76^ Ten accelerated stability studies assessed the storage of FDCs under market conditions of high temperatures and humidity, demonstrating effect on the dissolution rate and leading to rifampicin and ethambutol degradation. The formation of degradation products is of serious concern, especially when inactive against mycobacteria.^77^ It has been demonstrated in vitro that substandard drugs with degraded active ingredients select for gene alterations that confer resistance to standard TB medicines.^78^

Another unresolved issue is that in many low-income and middle-income countries, treatment of TB is left to the private sector,^79^ risking fuelling the MDR-TB epidemic by increasing inappropriate and indiscriminate antimicrobial use^80 81^ as medicines may be sold loose with no labelling or manufacturing information.^82^

Initiatives to improve medicine procurement as a means of reducing TB drug SF burden includes The WHO Prequalification of Medicines Programme, set up in 2001 to facilitate access to medicines that meet unified standards of quality, safety and efficacy. In 2023, 125 finished TB products were included in the list.

Nevertheless, as recently highlighted by Akpobolokemi *et al*^83^ surveillance of the quality of medicines needs to be an integral part of treatment programmes as they expand geographical coverage and use. Furthermore, new expensive antimicrobials, such as linezolid, bedaquiline, pretomanid and delamanid have now being deployed to optimise treatment of MDR-TB and they risk being falsified.

How SF anti-TB medicines impact on patient outcome and AMR and how important they should be ranked among other AMR drivers, such as poor adherence and poor prescribing, in different communities and HIV prevalences remains uncertain.^84^ Better understanding of the pharmacokinetic-pharmacodynamic relationships between anti-TB medicine concentrations and patient outcome and AMR^85^ are needed to understand the impact of SF anti-TB medicines.

In 2009, the WHO’s Beijing call for action made a commitment to accelerate efforts to prevent MDR/XDR-TB through effective TB care and control, and to scale up the diagnosis and treatment of MDR/XDR-TB.^32 86^ Nine years later, in September 2018, the first United Nations High-Level Meeting (UNHLM) on TB was held, where heads of state and governments committed to ending the TB epidemic by 2030.^11^ In order to achieve the targets and goals of the Sustainable Development Goals and the WHO’s End TB Strategy adopted in 2015,^68^ it will be vital to ensure that TB medicines are good quality or the gains observed in the last decade could be reversed as resistance threatens the improvements made.

## Conclusion

Poor-quality medicines pose a severe global public health threat but there is an insufficient evidence base to estimate the global prevalence of SF anti-TB medicines. The current global situation remains unclear, poorly documented, and the impact of SF medicines on engendering TB drug resistance uncertain.^83^ Only a handful of surveys have focused on specific drugs and in particular countries, and more information collected with standardised methodology is needed to compare between countries and regions. Although the curated data suggest an important issue with substandard anti-TB medicines and much less evidence for falsification, a large proportion of samples could not be classed into either category in the absence of packaging analysis/regulatory status data.

More research is needed on the development and evaluation of rapid, affordable and accurate portable devices to empower pharmacy inspectors for screening anti-TB medicines for those with %API and dissolution outside of reference limits.^87^

Important insights into the relationships between poor quality medicines of reduced %API/dissolution and patient outcome could be informed by evidence from TB medicine PK-PD relationships, especially the relationship between mg/kg body weight dosage variability and patient outcome.^88–94^

Surveillance of the quality of TB medicines needs to be an integral part of treatment programmes, an issue that should be taken into consideration at the next 2023 UNHLM on TB.

## Data Availability

Data are available in a public, open access repository.
