# Supplementary material for: Estimating the prevalence of poor-quality anti-TB medicines: a neglected risk for global TB control and resistance
Source: BMJ Glob Health. 2023 Jul 11;8(7):e012039. doi: 10.1136/bmjgh-2023-012039 (PMC10347509; doi:10.1136/bmjgh-2023-012039)
Supplement: Supplementary data [file bmjgh-2023-012039supp001.pdf]

SUPPLEMENTARY MATERIAL

Figure 1 PRIMA flow diagram of the selection process of the publications on anti-tuberculosis medicine quality. PRISMA, Preferred Reporting Items for Systematic Reviews and Meta-Analyses.

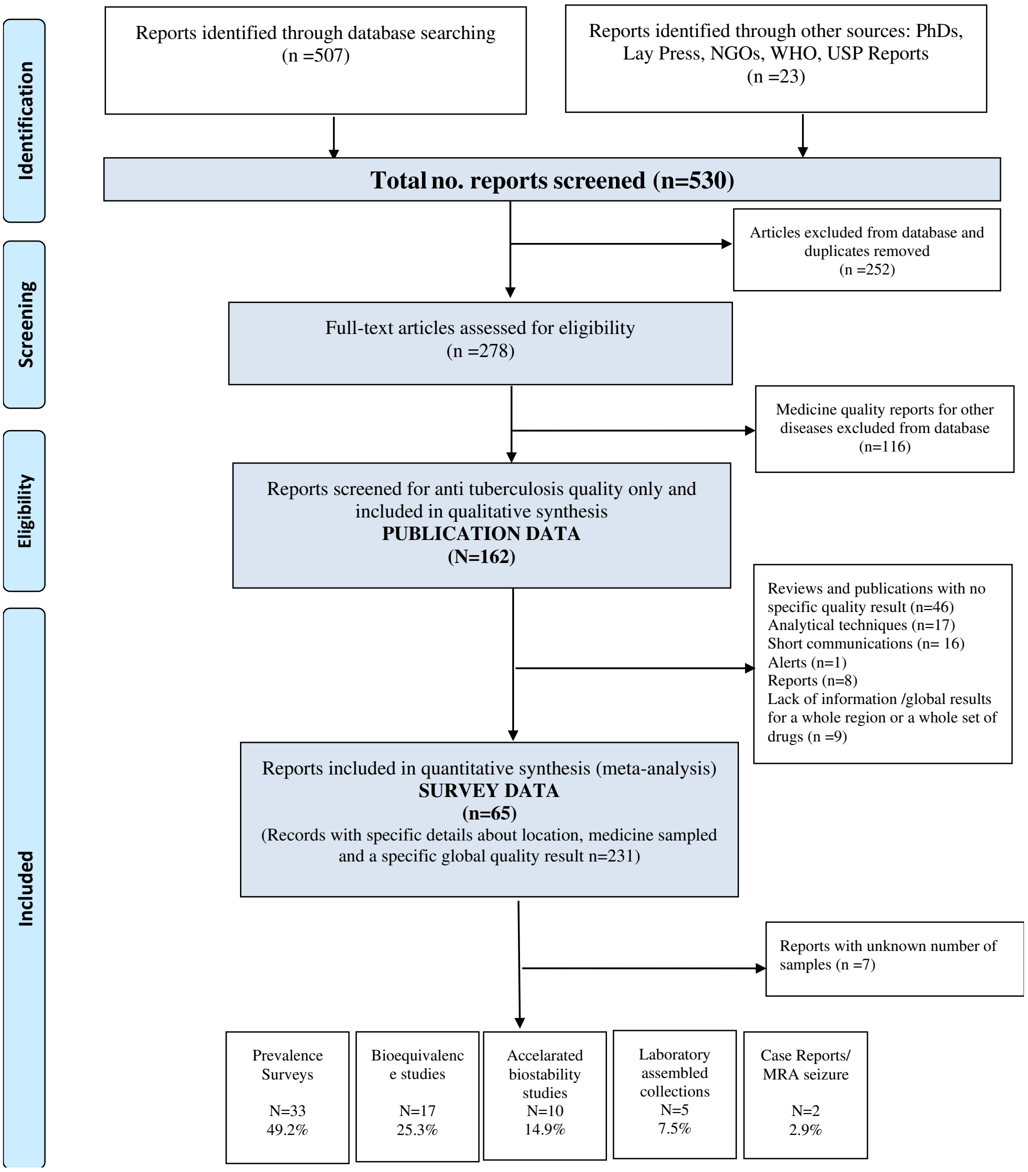

Supplementary Table 1. Search terms used for retrieving published reports with anti-tuberculosis medicine quality data.

English	French	Spanish
Falsified<br>fake<br>Substandard<br>Counterfeit<br>Spurious	Falsifié<br>Faux<br>De qualité inférieure<br>Contrefaçon<br>Spurious	Falsificado<br>Falso<br>Subestándar<br>Falsificación
Tuberculosis<br>Anti-tuberculosis<br>Tuberculosis medicines<br>Tuberculosis drugs.	Tuberculose<br>Anti-tuberculeux<br>Médicaments contre la tuberculose	Tuberculosis<br>Anti-tuberculosis<br>Medicinas Tuberculosis<br>Tratamiento Tuberculosis
Quality	Qualité	Calidad

Supplementary Table 2. Main characteristics of prevalence surveys, equivalence studies, accelerated biostability studies, case reports and laboratory assembled collections included in the review.

First Author	Year	Country	Medicine	Quality	Failure Rate	N	n failed
Convenience Survey							
Abuga, K.O.	2013	Kenya	Levofloxacin	Good Quality	0	2	0
			Meropenem	Good Quality	0	5	0
			Streptomycin	Good Quality	0	1	0
Agrawal, S.	2004	India	Pyrazinamide	Good Quality	0	1	0
			Rifampicin-Isoniazid-Pyrazinamide- Ethambutol	Good Quality	0	1	0
			Rifampicin	Good Quality	0	1	0
			Isoniazid	Good Quality	0	1	0
Ashokraj, Y.	2004	India	Rifampicin-Isoniazid-Pyrazinamide- Ethambutol	Good Quality	0	4	0
Ashokraj, Y.	2005	India	Rifampicin- Isoniazid- Ethambutol	SorF	50	44	22
Ashokraj, Y.	2006	India	Rifampicin-Isoniazid	Good Quality	0	2	0
			Rifampicin-Isoniazid-Pyrazinamide- Ethambutol	Good Quality	0	2	0
Awofisayo, S.O.	2010	Nigeria	Ofloxacin	Good Quality	0	9	0
Bate, R.	2013	India	Isoniazid	SorF	11	138	15
			Rifampicin	SorF	8	129	10

		Unknown - Angola, Brazil, China, Democratic Republic of Congo, Egypt, Ethiopia, Ghana, Kenya, Nigeria, Russia, Rwanda, Thailand, Turkey, Uganda, Tanzania, Zambia	Rifampicin	SorF	3	143	4
				SorF	16	103	16
			Isoniazid	SorF	4	140	6
				SorF	16	60	10
Bate, R.	2009	India	Rifampicin	SorF	6	52	3
			Isoniazid	SorF	17	48	8
			Isoniazid	SorF	6	36	2
			Rifampicin	SorF	12	66	8
Bate, R.	2014	India	Isoniazid	Falsified	2	166	3
		India	Isoniazid	Substandard	5		8
		Unknown - 18 countries	Rifampicin	Falsified	2	103	2
		Unknown - 18 countries	Rifampicin	Substandard	17		17
		India	Rifampicin	Falsified	2	167	3

		India	Rifampicin	Substandard	8		13
		Unknown - 18 countries	Isoniazid	Falsified	9	57	5
		Unknown - 18 countries	Isoniazid	Substandard	7		4
Bate, R.	2011	Unknown - 17 countries	Rifampicin	SorF	12	168	20
			Isoniazid	SorF	16	146	23
CDSCO. Central Drugs Standard Control Organization	2009	India	Rifampicin-Isoniazid-Pyrazinamide- Ethambutol	Good Quality	0	55	0
			Rifampicin-Isoniazid	Good Quality	0	110	0
			Pyrazinamide	Good Quality	0	80	0
Islam, M.R.	2017	Cambodia	Levofloxacin	SorF	30	60	18
			Ofloxacin	SorF	23	57	13
Kenyon, T.A.	1999	Botswana	Rifampicin-Isoniazid-Pyrazinamide	Substandard	17	6	1
			Rifampicin-Isoniazid	Substandard	20	5	1
			Rifampicin-Isoniazid-Pyrazinamide- Ethambutol	Substandard	100	2	2
Kibwage, I.O.	1992	Kenya	Streptomycin	SorF	100	2	2
		Kenya	Rifampicin	Good Quality	0	1	0
Kibwage, I.O.	1999	Kenya	Pyrazinamide	Good Quality	0	1	0
			Ethambutol	SorF	33	3	1
			Isoniazid	SorF	100	1	1
			Rifampicin	SorF	50	2	1
Laserson, K.F.	2001	India	Rifampicin-Isoniazid-Pyrazinamide- Ethambutol	SorF	14	14	2
			Isoniazid	Good Quality	0	10	0
			Rifampicin	SorF	13	16	2
Milán-Segovia, R. C.	2010	Mexico	Rifampicin-Isoniazid-Pyrazinamide	Good Quality	0	18	0
Mirani, H.	2007	India	Rifampicin	Falsified	100	Unkown	Unkown

			Streptomycin	Falsified	100	Unkown	Unkown
			Isoniazid	Falsified	100	Unkown	Unkown
Mweemba, W.	2011	Zambia	Rifampicin-Isoniazid-Pyrazinamide- Ethambutol	SorF	20	5	1
			Rifampicin-Isoniazid	SorF	14	7	1
			Rifampicin- Isoniazid- Ethambutol	Good Quality	0	5	0
Pouplin, T.	2014	Vietnam	Rifampicin-Isoniazid-Pyrazinamide	Good Quality	0	10	0
			Rifampicin	SorF	36	36	13
			Pyrazinamide	SorF	55	36	20
			Rifampicin	SorF	47	30	14
			Isoniazid	SorF	17	30	5
			Pyrazinamide	SorF	3	30	1
			Isoniazid	SorF	50	36	18
Prasad, B.	2008	India	Isoniazid-Ethambutol	SorF	40	5	2
			Rifampicin-Isoniazid-Pyrazinamide-Ethambutol-Pyridoxine	Good Quality	0	1	0
			Rifampicin-Isoniazid-Pyrazinamide-Ethambutol	SorF	25	4	1
			Rifampicin-Isoniazid-Ethambutol	SorF	40	10	4
			Ethambutol	SorF	33	15	5
Ramachandran, G.	2013	India	Ethambutol	Substandard	3	440	13
			Isoniazid	Substandard	4	406	16
			Rifampicin	Substandard	10	478	48
			Pyrazinamide	Substandard	6	423	25
			Cycloserine	Substandard	77	70	54
			Ethionamide	Good Quality	0	64	0
			Levofloxacin	Substandard	13	67	9
Seear, M.	2011	India	Rifampicin	SorF	44	100	44
	2007	India	Rifampicin	Good Quality	0	13	0

South East Asian FIP-WHO Forum of Pharmaceutical Associations			Rifampicin-Isoniazid-Ethambutol	Good Quality	0	10	0
			Rifampicin-Isoniazid-Pyrazinamide-Ethambutol	Good Quality	0	11	0
Thoithi, G.N.	2002	Kenya	Norfloxacin	Good Quality	0	5	0
			Ethambutol	Good Quality	0	1	0
			Rifampicin	Good Quality	0	1	0
Thoithi, G.N.	2008	Kenya	Ethambutol	Good Quality	0	3	0
			Rifampicin-Isoniazid-Pyrazinamide-Ethambutol	Good Quality	0	2	0
			Rifampicin-Isoniazid-Pyrazinamide	Good Quality	0	5	0
			Rifampicin-Isoniazid	Good Quality	0	3	0
			Isoniazid	Good Quality	0	1	0
			Norfloxacin	Good Quality	0	1	0
			Pyrazinamide	Good Quality	0	1	0
			Kanamycin	Good Quality	0	1	0
van Crevel, R.	2002	Indonesia	Rifampicin	Good Quality	0	59	0
van Crevel, R.	2004	Indonesia	Rifampicin	SorF	33	12	4
World Health Organization	2011	Republic of Belarus	Rifampicin-Isoniazid	Good Quality	0	12	0
		Azerbaijan	Rifampicin-Isoniazid	Good Quality	0	3	0
		Kazakhstan	Ofloxacin	SorF	16	12	2
		Armenia	Kanamycin	Good Quality	0	12	0
		Azerbaijan	Kanamycin	Good Quality	0	6	0
		Republic of Belarus	Kanamycin	Good Quality	0	12	0
		Kazakhstan	Kanamycin	Good Quality	0	12	0
		Ukraine	Kanamycin	Good Quality	0	13	0
		Uzbekistan	Kanamycin	Good Quality	0	12	0
		Armenia	Ofloxacin	SorF	8	12	1

		Republic of Belarus	Ofloxacin	SorF	25	12	3
		Ukraine	Ofloxacin	Good Quality	0	10	0
		Uzbekistan	Ofloxacin	Good Quality	0	3	0
		Uzbekistan	Rifampicin	SorF	43	7	3
		Kazakhstan	Isoniazid	SorF	8	12	1
		Kazakhstan	Rifampicin	SorF	83	12	10
		Azerbaijan	Isoniazid	SorF	16	12	2
		Republic of Belarus	Rifampicin	Good Quality	0	12	0
		Uzbekistan	Rifampicin-Isoniazid	Good Quality	0	12	0
		Azerbaijan	Rifampicin	SorF	16	6	1
		Kazakhstan	Rifampicin-Isoniazid	SorF	8	12	1
		Armenia	Rifampicin-Isoniazid	Good Quality	0	3	0
		Azerbaijan	Ofloxacin	Good Quality	0	4	0
		Armenia	Isoniazid	Good Quality	0	3	0
		Republic of Belarus	Isoniazid	SorF	8	12	1
		Ukraine	Isoniazid	SorF	10	19	2
		Armenia	Rifampicin	SorF	25	12	3
		Ukraine	Rifampicin	Good Quality	0	11	0
		Uzbekistan	Isoniazid	SorF	27	11	3
Random Survey							
Government of India	2017	India	Pyrazinamide	SorF	5	Unkown	0
			Amikacin	SorF	43	60	26
			Amikacin	SorF	19	164	31
Nabirova, D.	2017	Kazakhstan	Ofloxacin	SorF	46	26	12
			Moxifloxacin	Good Quality	0	40	0

			Rifampicin	SorF	29	176	51
			Isoniazid	SorF	7	72	5
			Levofloxacin	Good Quality	0	32	0
			Pyrazinamide	SorF	4	112	4
			Prothionamide	Good Quality	0	100	0
			Cycloserine	Good Quality	0	80	0
			Ethionamide	SorF	55	22	12
			Kanamycin	SorF	33	12	4
			Ethambutol	SorF	67	110	74
Rookkapan, K.	2005	Thailand	Pyrazinamide	SorF	26	46	12
			Isoniazid	Good Quality	0	51	0
			Ethambutol	SorF	14	51	7
			Rifampicin	SorF	62	50	31
Taylor, R.B.	2001	Nigeria	Isoniazid	SorF	100	4	4
			Rifampicin	SorF	33	15	5
			Pyrazinamide	SorF	100	3	3
			Streptomycin	SorF	53	19	10
World Health Organization	1999	Myanmar/Burma	Rifampicin	SorF	9	13	1
		Vietnam	Rifampicin	SorF	29	31	9
Bioavailability studies							
Agrawal, S.	2002	India	Isoniazid	Good Quality	0	13	0
			Rifampicin-Isoniazid-Pyrazinamide-Ethambutol	Good Quality	0	13	0
			Rifampicin	Good Quality	0	13	0
			Pyrazinamide	Good Quality	0	13	0
Agrawal, S.	2004	India	Rifampicin	Good Quality	0	22	0
			Isoniazid	Good Quality	0	22	0
			Rifampicin-Isoniazid-Pyrazinamide-Ethambutol	Good Quality	0	22	0

			Pyrazinamide	Good Quality	0	22	0
Ashokraj, Y.	2004	India	Rifampicin-Isoniazid-Pyrazinamide-Ethambutol	Good Quality	0	4	0
Ashokraj, Y.	2005	India	Rifampicin-Isoniazid-Ethambutol	Good Quality	0	44	0
Awofisayo, S.O.	2010	Nigeria	Ofloxacin	SorF	55	9	5
Garnham, J.C.	1976	United Kingdom	Rifampicin-Isoniazid	Good Quality	0	2	0
			Isoniazid	Good Quality	0	2	0
			Rifampicin	Good Quality	0	1	0
McIlleron, H.	2002	South Africa	Rifampicin-Isoniazid	Good Quality	0	15	0
			Isoniazid	Good Quality	0	118	0
			Pyrazinamide	Good Quality	0	118	0
			Ethambutol	Good Quality	0	113	0
			Streptomycin	Good Quality	0	64	0
			Ethionamide	Good Quality	0	1	0
			Rifampicin	Substandard	51	103	53
McIlleron, H.	1999	South Africa	Rifampicin-Isoniazid-Pyrazinamide-Ethambutol	Good Quality	0	47	0
			Rifampicin-Isoniazid-Pyrazinamide	Good Quality	0	19	0
Milán-Segovia, R. C.	2010	Mexico	Rifampicin-Isoniazid-Pyrazinamide	SorF	100	18	18
Panchagnula, R.	1999	India	Rifampicin-Isoniazid-Pyrazinamide	Good Quality	0	20	0
			Rifampicin	Good Quality	0	20	0
Panchagnula, R.	2003	India	Pyrazinamide	Good Quality	0	42	0
			Rifampicin-Isoniazid-Pyrazinamide	Good Quality	0	20	0
			Isoniazid	Good Quality	0	42	0
			Rifampicin-Isoniazid-Pyrazinamide-Ethambutol	Good Quality	0	22	0
Panchagnula, R.	2003	India	Rifampicin-Isoniazid-Pyrazinamide	Good Quality	0	1	0
			Rifampicin-Isoniazid-Pyrazinamide-Ethambutol	Good Quality	0	1	0
Pillai, G.	2001	South Africa	Rifampicin	Good Quality	0	10	0

Pillai, G.	1999	South Africa	Rifampicin-Isoniazid	SorF	67	3	2
			Rifampicin-Isoniazid-Pyrazinamide-Ethambutol	SorF	100	2	2
			Rifampicin-Isoniazid-Pyrazinamide	SorF	60	5	3
Shishoo, C.J.	2001	India	Rifampicin-Isoniazid	SorF	100	6	6
van Crevel, R.	2002	Indonesia	Rifampicin	SorF	70	62	43
van Crevel, R.	2004	Indonesia	Rifampicin	SorF	33	12	4
Accelarated biostability studies							
Ashokraj, Y.	2005	India	Rifampicin-Isoniazid-Pyrazinamide-Ethambutol	SorF	25	4	1
			Rifampicin-Isoniazid	Good Quality	0	2	0
Ashokraj, Y.	2004	India	Rifampicin-Isoniazid-Pyrazinamide-Ethambutol	Good Quality	0	4	0
Bhutani, H.	2004	India	Isoniazid-Ethambutol	SorF	100	1	1
Bhutani, H.	2004	India	Rifampicin-Isoniazid-Pyrazinamide-Ethambutol	SorF	100	3	3
		India	Rifampicin-Isoniazid-Pyrazinamide	SorF	100	2	2
		India	Rifampicin-Isoniazid	SorF	100	3	3
		India	Rifampicin-Isoniazid-Ethambutol	SorF	100	3	3
Bhutani, H.	2003	India	Ethambutol	SorF	33	3	1
			Isoniazid-Ethambutol	SorF	71	7	5
			Rifampicin-Isoniazid-Ethambutol	SorF	60	5	3
			Rifampicin-Isoniazid-Pyrazinamide-Ethambutol	SorF	29	7	2
Jaruratanasirikul, S.	2003	Thailand	Meropenem	Good Quality	0	12	0
Keel, R. A.	2011	USA	Meropenem	Good Quality	0	6	0
			Imipenem-Cilastatin	Good Quality	0	6	0
			Doripenem	Good Quality	0	6	0
Rao, K.V.N.	1968	India	Cycloserine	Good Quality	0	92	0
			Cycloserine	SorF	100	57	57
Singh, S.	2003	India	Rifampicin-Isoniazid-Pyrazinamide-Ethambutol	SorF	50	4	2
Singh, S.	2002	India	Isoniazid	Good Quality	0	1	0

			Rifampicin-Isoniazid	Good Quality	0	1	0
			Ethambutol	SorF	100	1	1
			Rifampicin-Isoniazid-Pyrazinamide	Good Quality	0	1	0
			Rifampicin-Isoniazid-Pyrazinamide-Ethambutol	SorF	100	1	1
			Isoniazid-Ethambutol	SorF	100	1	1
			Rifampicin-Isoniazid-Ethambutol	SorF	100	1	1
			Rifampicin	Good Quality	0	1	0
			Pyrazinamide	Good Quality	0	1	0
Case Reports/ MRA recalls confiscations							
Bernier, M. C.	2016	Unknown - Africa	Gatifloxacin /Ciprofloxacin found in Artemether–lumefantrine (AL)	Falsified	100	65	65
Braquehais, S.	2016	Cote d Ivoire	Rifampicin-Isoniazid-Pyrazinamide- Ethambutol	SorF	100	2 million doses	2 million doses
Caudron, J.M.	2008	Chad	Rifampicin	SorF	100	Unkown	Unkown
Health Canada	2009	Canada	Rifampicin	Substandard	100	Unkown	Unkown
Power, G.	2002	Vietnam	Ceftazidime	SorF	100	Unkown	Unkown
Sidley, P.	2008	South Africa	Rifampicin-Isoniazid-Pyrazinamide- Ethambutol	Substandard	100	Unkown	Unkown
South East Asian FIP-WHO Forum of Pharmaceutical Associations	2004	India	Ethambutol	SorF	100	1	1
			Rifampicin-Isoniazid	SorF	100	1	1
Laboratory Assembled Collections							
Agrawal, S.	2004	India	Rifampicin-Isoniazid-Pyrazinamide	Good Quality	0	1	0
			Rifampicin-Isoniazid-Pyrazinamide-Ethambutol	Good Quality	0	5	0
Ellard, G.A.	1999	United Kingdom	Rifampicin-Isoniazid	Good Quality	0	5	0
			Rifampicin-Isoniazid-Pyrazinamide	Good Quality	0	7	0
			Rifampicin-Isoniazid-Pyrazinamide-Ethambutol	Good Quality	0	2	0
Nguyen, D. T.	2008	Switzerland	Rifampicin-Isoniazid-Pyrazinamide	Good Quality	0	4	0
Panchagnula, R.	2006	India	Rifampicin-Isoniazid-Pyrazinamide- Ethambutol	Good Quality	0	2	0

			Rifampicin-Isoniazid-Pyrazinamide	Good Quality	0	2	0
			Rifampicin-Isoniazid	Good Quality	0	2	0
Zaheer, M.	2009	Pakistan	Ofloxacin	Good Quality	0	4	0
			Levofloxacin	SorF	25	4	1

Supplementary Table 3 Publications reviewed on analytical technique studies**.**

#	Title	First author	Year	Country	Techniques described	Type of technique	Reference
1	Comparative Assessment of the Quality Control Measurements of Multisource Ofloxacin Tablets Marketed in Nigeria	Awofisayo. S.O.	2010	Nigeria	Uniformity of weight. friability. and disintegration. Simulated gastric fluid (SGF) and simulated intestinal fluid (SIF) without enzymes methods were used to estimate dissolution rate and disintegration time. Dissolution efficiency (DE) and predicted availability equivalence (PAE) were used to estimate the likely in vivo bioavailability for ofloxacin	Bioavailability	(1)
2	In vitro evaluation of food effect on the bioavailability of rifampicin from antituberculosis fixed dose combination formulations	Panchagnula. R.	2003	India	In vitro effect of food on the bioavailability of rifampicin/Dissolution	Bioavailability and food	(2)
3	Bioequivalence trials of rifampicin containing formulations: extrinsic and intrinsic factors in the absorption of rifampicin	Agrawal. S.	2004	India	Study of extrinsic and intrinsic factors in the absorption of rifampicin	Bioavailability and food	(3)
4	Evaluation of bioequivalence of isoniazid and pyrazinamide in three and four drugs fixed dose combinations using WHO simplified protocol	Panchagnula. R.	2003	India	Evaluation of WHO simplified screening protocol for bioequivalence assessment of other medicines than rifampicin	Bioequivalence	(4)
5	Screening of bulk drug samples and anti-tuberculosis products for the presence of therapeutically less active diasteriomeric (R.S) form of ethambutol dihydrochloride	Prasad. B.	2008	India	Screening for the presence less active diasteriomeric (R.S) forms of ethambutol dihydrochloride. differential scanning calorimetric method.	Calorimetric	(5)
6	Automated chemiluminometric screening of counterfeit drugs of the antituberculosis agent pyrazinamide	Prior. J. A.	2009	Portugal	Development and validation of a chemiluminescent method for pyrazinamide	Chemiluminescent	(6)

7	Drug quality screening in developing countries: establishment of an appropriate laboratory in Swaziland	Kenyon. T.A.	1994	Swaziland	Establishment of a drug quality screening laboratory in Swaziland. Thin-Layer Chromatography	Chromatography	(7)
8	A Compendium of Unofficial Methods for Rapid Screening of Pharmaceuticals by Thin-Layer Chromatography	Kenyon. A.S. & Layloff. T.P.	1995	USA	Thin-Layer Chromatography	Chromatography	(8)
9	Liquid Chromatographic Separation of Isoniazid. Pyrazinamide and Rifampicin on a Reversed-Phase Silica Column	Thoithi. G.N.	2002	Kenya	A gradient liquid chromatographic method	Chromatography	(9)
10	Liquid chromatographic determination of isoniazid. pyrazinamide and rifampicin from pharmaceutical preparations and blood	Khuhawar. M. Y.	2002	Pakistan	Liquid Chromatography	Chromatography	(10)
11	Validation of an ultra-fast UPLC-UV method for the separation of antituberculosis tablets	Nguyen. D. T.	2008	Switzerland	Development and validation of a ultra-performance LC (UPLC) and HTUPLC method coupled with UV detection	Chromatography	(11)
12	Transfer of Minilab TLC Screening Methods to Quantitative HPTLC-Densitometry for Pyrazinamide. Ethambutol. Isoniazid. and Rifampicin in a Combination Tablet	Strock. J.	2015	USA	Minilab TLC Screening Methods to Quantitative HPTLC-Densitometry	Chromatography	(12)
13	Impaired bioavailability of rifampicin in presence of isoniazid from fixed dose combination (FDC) formulation	Shishoo. C.J.	2001	India	Development and validation of a HPTLC method for bioavailability studies of rifampicin and its metabolite 25-Desacetylrifampicin (25-DAR) in urine	Chromatograpy	(13)

14	The colorimetric analysis of anti-tuberculosis fixed-dose combination tablets and capsules	Ellard. G.A.	1999	UK	Colorimetric Methods	Colorimetry	(14)
15	A disintegration test for evaluation of drug availability from tablets and capsules	Sandell. E.	1970	Sweden	Disintegration test	Disintegration	(15)
16	Dissolution test as a surrogate for quality evaluation of rifampicin containing fixed dose combination formulations	Agrawal. S.	2004	India	Development of a dissolution methodology to predict in vivo performance of rifampicin	Dissolution	(16)
17	Dissolution testing of marketed rifampicin containing fixed dose combination formulations using a new discriminative media: a post marketing retrospective study	Panchagnula. R.	2006	UK	Validate a new method of dissolution testing for solid dosage forms of rifampicin containing formulations	Dissolution	(17)
18	A Decision Tree for Rapid Quality Assurance and Control of Rifampicin-Containing Oral Dosage Forms for Global Distribution for Tuberculosis Treatment	Ashokraj. Y.	2008	India	Dissolution test/ Bioequivalence analysis by plasma pooling	Dissolution	(18)
19	Paper Analytical Device To Detect Substandard Anti-tuberculosis Medications	Reiser. H.	2012	USA	Paper Analytical Devices (PAD)	PAD	(19)
20	Paper Analytical Devices for Fast Field Screening of Beta Lactam Antibiotics and Antituberculosis Pharmaceuticals	Weaver. A.A.	2013	USA	Paper Analytical Devices (PAD)	PAD	(20)
21	In vitro Analysis and Data Comparison of Market Brands of Ciprofloxacin. Ofloxacin and Levofloxacin	Zaheer. M.	2009	Pakistan	Study of the disintegration time. dissolution rate and assay by spectrophotometer and HPLC in quinolones	Spectroscopy	(21)

22	Dissolution testing of isoniazid. rifampicin. pyrazinamide and ethambutol tablets using near-infrared spectroscopy (NIRS) and multivariate calibration	de Oliveira Neves. A.C.	2012	Brazil	Near-infrared spectroscopy (NIRS) and multivariate calibration to measure the percentage drug dissolution	Spectroscopy	(22)
23	Spectroscopic Techniques for Nondestructive Quality Inspection of Pharmaceutical Products: A Review	Kandpal Lalit. M.	2015	Korea	Spectroscopic Techniques	Spectroscopy	(23)
24	Triboelectric Nanogenerator (TENG) Mass Spectrometry of Falsified Antimalarials	Bernier. M. C.	2018	USA	Triboelectric nanogenerator (TENG) mass spectrometry for ciprofloxacin/ gatifloxacin	Spectroscopy	(24)
25	Evaluating Low-Cost Optical Spectrometers for the Detection of Simulated Substandard and Falsified Medicines	Wang. W.	2020	USA	Low-Cost Optical Spectrometers	Spectroscopy	(25)

REFERENCES

1. Awofisayo SO, Awofisayo OA, Eyen N, Udoh IE. Comparative Assessment of the Quality Control Measurements of Multisource Ofloxacin Tablets Marketed in Nigeria. Dissolution Technologies. 2010:20-5.
2. Panchagnula R, Rungta S, Sancheti P, Agrawal S, Kaul CL. In vitro evaluation of food effect on the bioavailability of rifampicin from antituberculosis fixed dose combination formulations. Il Farmaco. 2003;58(11):1099-103.
3. Agrawal S, Singh I, Kaur KJ, Bhade S, Kaul CL, Panchagnula R. Bioequivalence trials of rifampicin containing formulations: extrinsic and intrinsic factors in the absorption of rifampicin. Pharmacological Research. 2004;50(3):317-27.
4. Panchagnula R, Sancheti P, Rungta S, Agrawal S, Kaul CL. Evaluation of bioequivalence of isoniazid and pyrazinamide in three and four drugs fixed dose combinations using WHO simplified protocol. Pharmacological Research. 2003;48(4):383-7.
5. Prasad B, Kumar V, Bhutani H, Singh S. Screening of bulk drug samples and anti-tuberculosis products for the presence of therapeutically less active diasteriomeric (R,S) form of ethambutol dihydrochloride. Indian J Tuberc. 2008;55(4):192-8.
6. Prior JA, Santos JL, Lima JL. Automated chemiluminometric screening of counterfeit drugs of the antituberculosis agent pyrazinamide. J AOAC Int. 2009;92(3):830-6.

7. Kenyon TA, Kenyon AS, Sibiya T. Drug quality screening in developing countries: establishment of an appropriate laboratory in Swaziland. Bull World Health Organ. 1994;72(4):615-20.
8. Kenyon AS, Layloff TP. A Compendium of Unofficial Methods for Rapid Screening of Pharmaceuticals by Thin-Layer Chromatography. Food and Drug Administration FDADivision of Testing and Applied Analytical Development. 1995
9. Thoithi GN, Kibwage IO, King'ondu O, Hoogmartens J. Liquid Chromatographic Separation of Isoniazid, Pyrazinamide and Rifampicin on a Reversed-Phase Silica Column. East and Central African Journal of Pharmaceutical Sciences. 2002;5(1).
10. Khuhawar MY, Rind FMA. Liquid chromatographic determination of isoniazid, pyrazinamide and rifampicin from pharmaceutical preparations and blood. Journal of Chromatography B. 2002;766(2):357-63.
11. Nguyen DT, Guillarme D, Rudaz S, Veuthey JL. Validation of an ultra-fast UPLC-UV method for the separation of antituberculosis tablets. J Sep Sci. 2008;31(6-7):1050-6.
12. Strock J, Nguyen M, Sherma J. Transfer of Minilab TLC Screening Methods to Quantitative HPTLC-Densitometry for Pyrazinamide, Ethambutol, Isoniazid, and Rifampicin in a Combination Tablet. Journal of Liquid Chromatography & Related Technologies. 2015;38(11):1126-30.
13. Shishoo CJ, Shah SA, Rathod IS, Savale SS, Vora MJ. Impaired bioavailability of rifampicin in presence of isoniazid from fixed dose combination (FDC) formulation. International Journal of Pharmaceutics. 2001;228(1–2):53-67.
14. Ellard GA. The colorimetric analysis of anti-tuberculosis fixed-dose combination tablets and capsules. Int J Tuberc Lung Dis. 1999;3(11 Suppl 3):S343-6; discussion S51-2.
15. Sandell E, Eriksson K, Mellström G. A disintegration test for evaluation of drug availability from tablets and capsules. Acta pharmaceutica Suecica. 1970;7(5):559-66.
16. Agrawal S, Panchagnula R. Dissolution test as a surrogate for quality evaluation of rifampicin containing fixed dose combination formulations. International Journal of Pharmaceutics. 2004;287(1–2):97-112.
17. Panchagnula R, Kumar Bajpai A, Agrawal S, Ashokraj Y. Dissolution testing of marketed rifampicin containing fixed dose combination formulations using a new discriminative media: a post marketing retrospective study. Pharmazie. 2006;61(10):851-4.
18. Ashokraj Y, Agrawal S, Panchagnula R. A decision tree for rapid quality assurance and control of rifampicin-containing oral dosage forms for global distribution for tuberculosis treatment. Indian J Pharm Sci. 2008;70(1):1-4.
19. Reiser H, Raddell K, Lewis K, Jensen B, Lieberman M, editors. Paper Analytical Device To Detect Substandard Anti-tuberculosis Medications. American Society of Tropical Medicine and Hygiene Meeting 2012; 2012 November 11-15th, 2012; Atlanta, GA, USA American Society of Tropical Medicine and Hygiene.
20. Weaver AA, Reiser H, Barstis T, Benvenuti M, Ghosh D, Hunckler M, et al. Paper analytical devices for fast field screening of beta lactam antibiotics and antituberculosis pharmaceuticals. Anal Chem. 2013;85(13):6453-60.
21. Zaheer M, Rahman S, Mahmood S, Saleem M. In vitro analysis and data comparison of market brands of ciprofloxacin, ofloxacin and levofloxacin. Biological Sciences-PJSIR. 2009;52(4):186-90.

22. de Oliveira Neves AC, Soares GM, de Morais SC, da Costa FSL, Porto DL, de Lima KMG. Dissolution testing of isoniazid, rifampicin, pyrazinamide and ethambutol tablets using near-infrared spectroscopy (NIRS) and multivariate calibration. Journal of Pharmaceutical and Biomedical Analysis. 2012;57:115-9.
23. Kandpal Lalit M, Park E, Tewari J, Cho B-K. Spectroscopic Techniques for Nondestructive Quality Inspection of Pharmaceutical Products: A Review. Journal of Biosystems Engineering. 2015;40(4):394-408.
24. Bernier MC, Li A, Winalski L, Zi Y, Li Y, Caillet C, et al. Triboelectric Nanogenerator (TENG) Mass Spectrometry of Falsified Antimalarials. Rapid Commun Mass Spectrom. 2018;32(18):1585-90.
25. Wang W, Keller MD, Baughman T, Wilson BK. Evaluating Low-Cost Optical Spectrometers for the Detection of Simulated Substandard and Falsified Medicines. Applied spectroscopy. 2020;74(3):323-33.
